# Crosslinker-Integrated Photocleavable Gelatin–PEG Hydrogel via Bioorthogonal SPAAC Chemistry for UV-Triggered On-Demand Degradation

**DOI:** 10.3390/ma19122625

**Published:** 2026-06-18

**Authors:** Yeon Tae Kang, Gayeon Pyo, Karthika Muthuramalingam, Hyun Jong Lee

**Affiliations:** School of Chemical, Biological and Battery Engineering, Gachon University, 1342 Seongnam-daero, Seongnam-si 13120, Gyeonggi-do, Republic of Korea

**Keywords:** photocleavable hydrogel, strain-promoted azide–alkyne cycloaddition (SPAAC), gelatin–PEG hybrid, UV-triggered degradation, o-nitrobenzyl

## Abstract

Light-triggered hydrogel systems offer precise spatiotemporal control over drug release, yet most existing approaches require direct chemical conjugation of a photocleavable linker to the payload, which risks compromising bioactivity and limits applicability to structurally diverse molecules. Here, we report a gelatin–poly(ethylene glycol) (PEG) hybrid hydrogel crosslinked via strain-promoted azide–alkyne cycloaddition (SPAAC) click chemistry, in which an o-nitrobenzyl photocleavable (PC) linker is incorporated into the PEG crosslinker arm rather than conjugated to the drug. Acetylated gelatin–azide (AGA) was synthesized by sequential azide functionalization and amine capping of gelatin, and four-arm PEG-PC-DBCO (4armPEG-PC-DBCO) was prepared by coupling a PC DBCO-PEG4-NHS ester to four-arm PEG amine. Successful incorporation of the azide, DBCO, and o-nitrobenzyl moieties was confirmed by FT-IR spectroscopy, ^1^H NMR spectroscopy, and UV-Vis spectrophotometry. Hydrogel formation under physiological conditions (PBS, 37 °C) without catalysts or initiators was verified by rheological frequency sweep analysis, which confirmed elastic-dominant behavior (G′ > G″). Upon irradiation at 365 nm, the crosslinker was cleaved, and rapid network dissolution was observed both macroscopically and by in situ time sweep rheology. This platform enables on-demand, UV-selective hydrogel degradation independently of payload identity, providing a versatile foundation for future controlled drug release applications and dynamic, on-demand degradable scaffolds for tissue engineering.

## 1. Introduction

Hydrogels are three-dimensional polymer networks with high water content that structurally resemble the extracellular matrix (ECM), endowing them with excellent biocompatibility and mechanical compliance [1]. Among the diverse polymer systems explored for hydrogel fabrication, gelatin-based materials have attracted considerable interest owing to their natural origin, biodegradability, and the abundance of reactive functional groups—including primary amines and carboxylic acids—that facilitate chemical modification and crosslinking [1]. These properties have established gelatin hydrogels as versatile platforms in tissue engineering, wound management, and controlled drug delivery [2]. However, most conventional hydrogel-based delivery systems rely on passive diffusion or matrix degradation for drug release, which constrains the ability to regulate the timing and location of therapeutic action with precision. This limitation has driven the development of stimuli-responsive hydrogels capable of releasing their payloads on demand in response to external or physiological cues [3].

Among the various stimuli explored—including pH, temperature, and enzymatic activity—light offers a distinct set of advantages as an external trigger [3,4]. Unlike internal physiological stimuli, which are inherently tied to the surrounding biological environment and can vary across individuals and disease states, light can be applied non-invasively from outside the body with precise spatial and temporal control. The irradiation site, duration, and intensity can be adjusted independently and with immediacy, enabling on-demand activation without lasting perturbation of the surrounding tissue [5]. Furthermore, the stimulus disappears instantly upon cessation of irradiation, reducing the risk of off-target effects. These characteristics make light-triggered systems particularly attractive for applications involving tissues accessible to external irradiation, such as skin wounds and the corneal surface, where spatiotemporally controlled delivery of bioactive agents may be desirable [5].

Photocleavable (PC) linkers based on the o-nitrobenzyl scaffold represent the most extensively employed light-responsive elements in hydrogel engineering, owing to their well-characterized photolysis mechanism and strong UV absorbance near 365 nm [6]. Upon irradiation, the o-nitrobenzyl bond undergoes irreversible cleavage to yield a carboxylic acid and an o-nitrosobenzaldehyde, enabling programmable modifications to hydrogel structure and function. This chemistry has been exploited through two distinct design strategies. In the first approach, PC linkers are conjugated directly to therapeutic molecules, tethering them within the hydrogel matrix so that UV irradiation liberates the drug in a spatiotemporally controlled manner [7,8]. In the second approach, PC linkers are incorporated into the crosslinks of the polymer network itself, such that photocleavage leads to network degradation and subsequent release of physically entrapped payloads [6,8]. Both strategies have demonstrated utility in on-demand drug delivery, and the choice between them carries significant implications for platform design and versatility.

Despite their promise, existing photocleavable hydrogel systems present two practical limitations that restrict their broader applicability. First, approaches that rely on direct conjugation of the PC linker to the therapeutic agent require that each drug undergo bespoke chemical modification prior to loading. This derivatization step introduces the risk of altering the drug’s tertiary structure, receptor-binding affinity, or pharmacological activity, and it restricts the platform to therapeutics that are chemically compatible with the conjugation chemistry. Second, many reported PC hydrogel systems use a photoinitiator-mediated crosslinking reaction—typically driven by visible or blue light—in combination with subsequent UV-triggered PC cleavage for drug release. The spectral overlap between these two wavelength ranges undermines the orthogonality of each step: blue-light exposure intended for crosslinking may inadvertently activate the PC linker, while UV irradiation for release may partially stimulate the photoinitiator, compromising the selectivity of both the gelation and release processes [7,9].

In this study, we address both limitations simultaneously through a material design strategy that combines strain-promoted azide–alkyne cycloaddition (SPAAC) crosslinking with the integration of o-nitrobenzyl PC linkers directly into the PEG crosslinker arm. SPAAC is a copper-free, bioorthogonal cycloaddition reaction between strained cyclooctynes and azides that proceeds spontaneously under physiological conditions without any photoinitiator or external energy source [10]. By eliminating the light-dependent crosslinking step entirely, UV irradiation can be reserved exclusively as a trigger for PC linker cleavage, restoring full wavelength selectivity. Moreover, because the PC linker is located within the crosslinking junction rather than on the drug molecule, UV-induced network degradation releases encapsulated substances in their chemically unmodified form. This design is, in principle, compatible with a broad range of payloads—including pharmaceutical agents, growth factors, and cosmetic active ingredients—without requiring any payload-specific modification. To demonstrate this strategy, we report the synthesis of acetylated gelatin–azide (AGA) and 4armPEG-PC-DBCO, their crosslinking via SPAAC to form a photocleavable hydrogel, and the chemical and rheological characterization of the resulting material, including its UV-triggered degradation behavior.

## 2. Materials and Methods

### 2.1. Materials

Gelatin (type A, from porcine skin), acetic anhydride, sodium bicarbonate (NaHCO_3_), dimethyl sulfoxide (DMSO), and phosphate-buffered saline (PBS) were purchased from Sigma-Aldrich (St. Louis, MO, USA). NHS-PEG4-azide and DBCO-PEG4-NHS ester were obtained from Click Chemistry Tools (Scottsdale, AZ, USA). PC DBCO-PEG4-NHS ester (o-nitrobenzyl photocleavable linker) was purchased from BroadPharm (San Diego, CA, USA). Four-arm PEG amine (4armPEG-NH_2_, 10 kDa) was obtained from JenKem Technology (Allen, TX, USA). All reagents were used as received unless otherwise stated.

### 2.2. Synthesis of Acetylated Gelatin–Azide (AGA)

Gelatin (0.16 g) was dissolved in PBS (2 mL) at 37 °C. NHS-PEG4-azide (1080 μL, 100 mg/mL in PBS) was added and the mixture was reacted at 37 °C overnight to introduce azide groups onto the primary amines of gelatin via NHS ester chemistry, yielding gelatin–azide (GA). The product was purified by dialysis against PBS for 24 h (7000 Da MWCO) and lyophilized.

To cap remaining primary amines, GA was redissolved in DI water and the pH was adjusted to 8.0–8.5 with 0.1 M NaHCO_3_. Acetic anhydride was added dropwise at a 3:1 molar ratio relative to the primary amine content and stirred at room temperature for 1 h. The product was dialyzed against DI water for 24 h and lyophilized to yield acetylated gelatin–azide (AGA).

### 2.3. Synthesis of PEG Crosslinkers

4armPEG-NH_2_ was dissolved in a mixture of 90% DMSO/10% 0.1 M NaHCO_3_ (*v*/*v*) at 100 mg/mL. For 4armPEG-PC-DBCO, PC DBCO-PEG4-NHS ester (1000 μL, 100 mg/mL in DMSO) was added at a 6-fold molar excess relative to the amine groups of 4armPEG-NH_2_ (360 μL) and reacted at room temperature overnight. For 4armPEG-DBCO, which served as a spectroscopic reference lacking the photocleavable linker, DBCO-PEG4-NHS ester (700 μL, 100 mg/mL in DMSO) was used in place of the PC variant under otherwise identical conditions. Both products were purified by dialysis against DI water for 24 h (2000 Da MWCO) and lyophilized.

### 2.4. Hydrogel Formation

AGA and 4armPEG-PC-DBCO were each dissolved in PBS at the desired concentration. Hydrogels were formed by mixing equal volumes (1:1 *v*/*v*) of the two precursor solutions at room temperature. Gelation proceeded via strain-promoted azide–alkyne cycloaddition (SPAAC) between the azide groups of AGA and the DBCO groups of 4armPEG-PC-DBCO without the need for a catalyst or additional reagents.

### 2.5. Chemical Characterization

FT-IR spectroscopy. Fourier-transform infrared spectra were collected using an iS50 spectrometer (Thermo Fisher Scientific, Waltham, MA, USA) in attenuated total reflectance (ATR) mode over the range of 400–4000 cm^−1^. Lyophilized samples of gelatin, GA, AGA, 4armPEG-NH_2_, 4armPEG-DBCO, and 4armPEG-PC-DBCO were analyzed.

^1^H NMR spectroscopy. ^1^H NMR spectra were recorded on a JNM-ECZ500R/S1 spectrometer (JEOL, Tokyo, Japan) at 500 MHz. Gelatin-based samples (gelatin, GA, AGA) were dissolved in D_2_O; PEG-based samples (4armPEG-NH_2_, 4armPEG-DBCO, 4armPEG-PC-DBCO) were dissolved in DMSO-d_6_. Chemical shifts are reported in ppm.

UV-Vis absorption spectra of 4armPEG-DBCO and 4armPEG-PC-DBCO (0.5 mg/mL in 90% DMSO/10% 0.1 M NaHCO_3_ (*v*/*v*)) were measured from 250 to 400 nm using a V-770 spectrophotometer (Jasco, Tokyo, Japan) at the Smart Materials Research Center for IoT, Gachon University, Republic of Korea. The DBCO content of 4armPEG-DBCO was quantified from the baseline-corrected absorbance at 309 nm using the Beer–Lambert law, applying the molar extinction coefficient of DBCO (ε_309_ = 12,000 M^−1^cm^−1^).

### 2.6. Rheological Characterization

Viscoelastic properties of the hydrogels were evaluated using a MCR 92 rheometer (Anton Paar, Graz, Austria) with a parallel plate geometry at 37 °C. Frequency sweep measurements were performed over the range of 0.1–10 Hz at a constant strain of 1% to determine storage modulus (G′) and loss modulus (G″). To monitor real-time gelation kinetics, time sweep measurements were conducted at 1% strain and 1 Hz at 37 °C using a UV-transparent quartz plate. UV irradiation at 365 nm was initiated at 60 s to trigger photocleavage of the crosslinker during the measurement. The rheological data presented are representative curves obtained from independently prepared hydrogel batches, which exhibited consistent gelation and degradation behavior.

### 2.7. UV-Triggered Photocleavage

UV irradiation of hydrogel samples was performed using a 365 nm UV lamp (AS00-4H-TP, Altis, Ansan, Republic of Korea) operated at 80% output (manufacturer-specified maximum irradiance: 5980 mW/cm^2^ at a working distance of 12 mm and a spot diameter of 6 mm). To visualize UV-induced degradation, photographs of the hydrogel were taken at 30 s intervals during continuous irradiation.

## 3. Results

**Figure 1:**
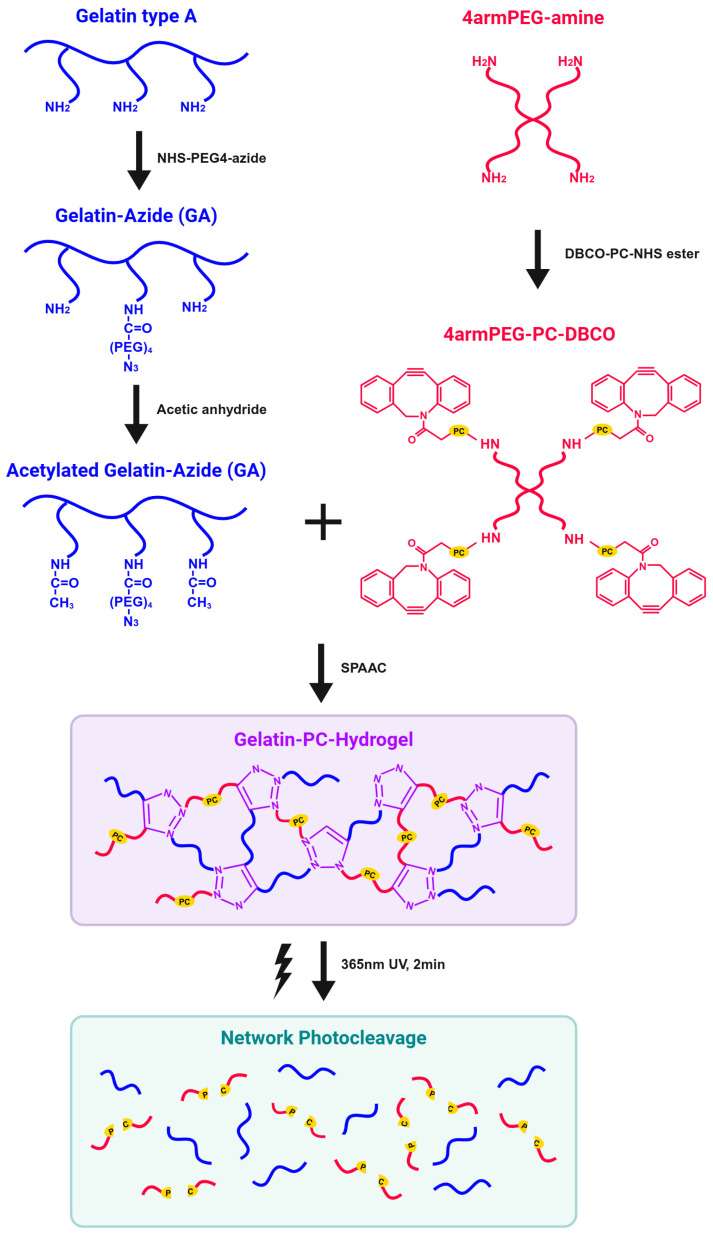
Synthesis scheme and design concept of the photocleavable gelatin–PEG hydrogel. Gelatin type A was sequentially modified to produce acetylated gelatin–azide (AGA), while 4armPEG-NH_2_ was functionalized with a PC DBCO-PEG4-NHS ester to yield 4armPEG-PC-DBCO, in which an o-nitrobenzyl photocleavable linker is incorporated at each DBCO-bearing arm terminus (dashed lines indicate the cleavable benzylic C–O bond). AGA and 4armPEG-PC-DBCO were crosslinked via strain-promoted azide–alkyne cycloaddition (SPAAC) without any photoinitiator or light source to form a stable hydrogel network, which undergoes selective degradation upon 365 nm UV irradiation through photocleavage of the o-nitrobenzyl linker at the crosslinking junctions, which may enable release of physically entrapped payloads.

**Figure 2:**
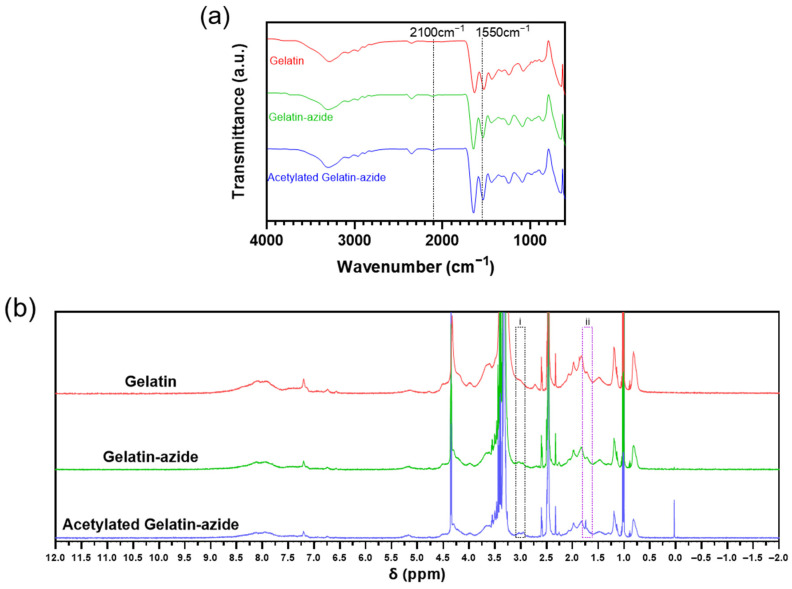
FT-IR and ^1^H NMR characterization of gelatin type A, gelatin–azide (GA), and acetylated gelatin–azide (AGA). (**a**) In the FT-IR spectra, the appearance of an azide stretching band at 2100 cm^−1^ in GA and AGA confirms successful azide conjugation via NHS-PEG4-azide coupling, and the amide II band at 1550 cm^−1^ reflects amide bond formation through the NHS ester coupling reaction. (**b**) In the ^1^H NMR spectra, the progressive attenuation of the signal at ~3 ppm (Box i), attributed to the methylene protons of lysine residues adjacent to the primary amine, confirms the stepwise consumption of free amine groups through azide conjugation and acetylation, while the peak at ~1.9 ppm (Box ii) becomes significantly more prominent in AGA, consistent with the contribution of acetyl methyl protons introduced by acetic anhydride treatment.

**Figure 3:**
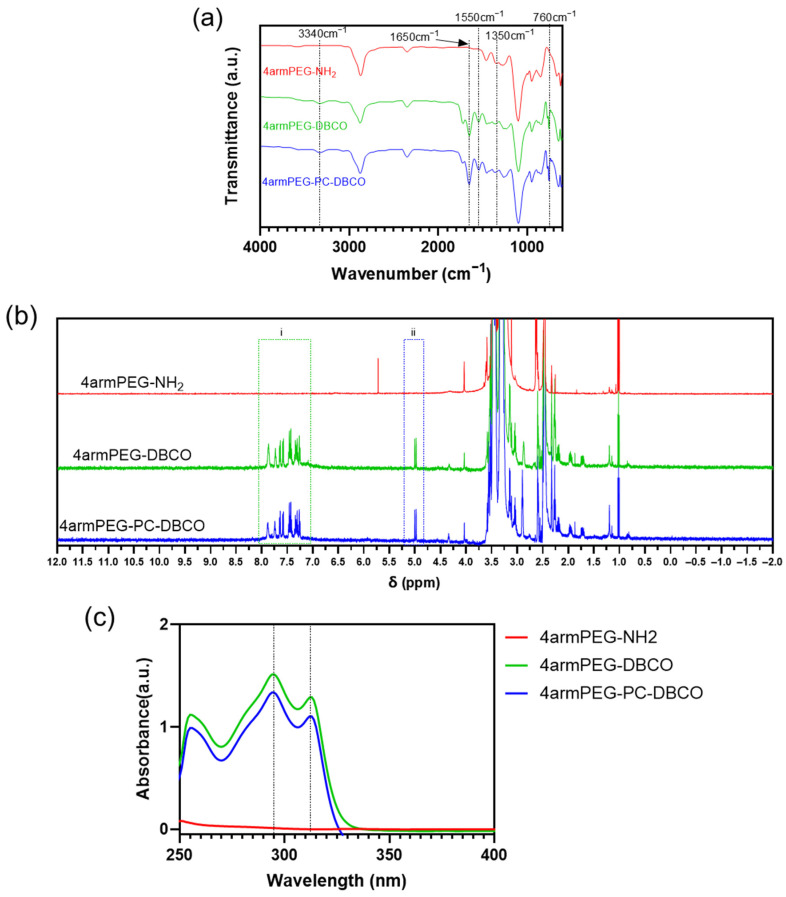
FT-IR, ^1^H NMR, and UV-Vis characterization of 4armPEG-NH_2_, 4armPEG-DBCO, and 4armPEG-PC-DBCO. (**a**) In the FT-IR spectra, the appearance of amide N–H stretching (3340 cm^−1^), amide I (1650 cm^−1^), and amide II (1550 cm^−1^) bands in both modified PEGs confirms amide bond formation via NHS ester coupling, while the additional bands at 1760 cm^−1^ (carbonate C=O) and 1350 cm^−1^ (N=O stretching) in 4armPEG-PC-DBCO confirm incorporation of the PC linker. (**b**) In the ^1^H NMR spectra, aromatic signals in Box i (~7–8.5 ppm) and aliphatic CH signals in Box ii (~4.5–5.5 ppm) confirm DBCO conjugation in both derivatives, with 4armPEG-PC-DBCO showing additional signals from the o-nitrobenzyl moiety. (**c**) UV-Vis spectra confirm DBCO incorporation through characteristic absorptions at ~290 and ~310 nm in both modified PEGs, absent in 4armPEG-NH_2_.

**Figure 4:**
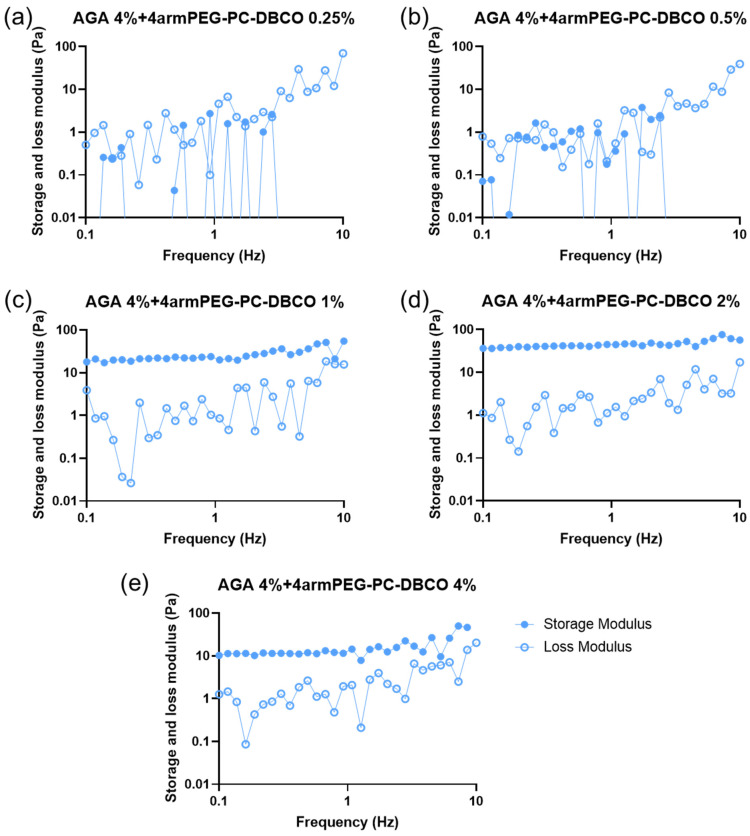
Oscillatory frequency sweep rheology of hydrogels formed with 4% (*w*/*v*) AGA combined with 4armPEG-PC-DBCO at (**a**) 0.25%, (**b**) 0.5%, (**c**) 1%, (**d**) 2%, and (**e**) 4% (*w*/*v*). Storage modulus (G′, filled circles) and loss modulus (G″, open circles) are shown as a function of frequency (0.1–10 Hz). Measurements were performed at 1% strain and 37 °C. Gel formation, defined as G′ > G″ across the measured frequency range, was observed at 4armPEG-PC-DBCO concentrations of 1% and above, with the 2% formulation yielding the highest G′ (~44.6 Pa at 1 Hz).

**Figure 5:**
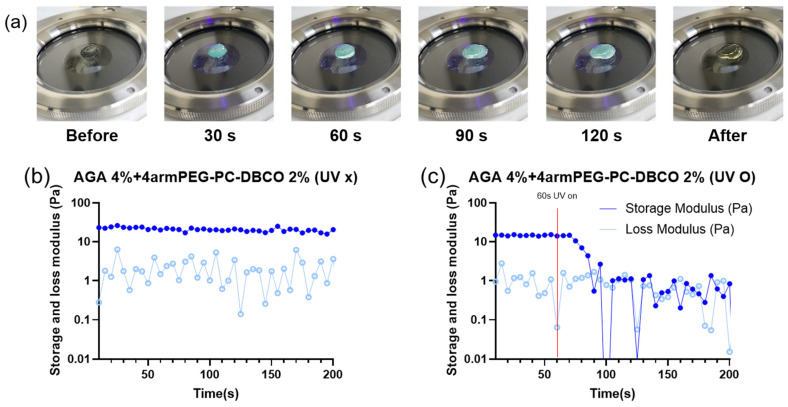
UV-triggered photocleavage behavior of the PC hydrogel (4% AGA/2% 4armPEG-PC-DBCO). (**a**) Macroscopic photographs of the hydrogel recorded at 30 s intervals before, during, and after 365 nm UV irradiation (total irradiation: 120 s). (**b**) Time-sweep rheology without UV irradiation (UV(x)), showing stable G′ (filled circles) and G″ (open circles) throughout the 200 s measurement. (**c**) Time-sweep rheology of the hydrogel with UV irradiation (UV(o)) initiated at 60 s (red vertical line), showing rapid decline of G′ below G″ within approximately 20 s of UV onset, followed by irreversible loss of the gel state. UV(o) and UV(x) denote the presence and absence of UV irradiation, respectively. All measurements were performed at 1% strain, 1 Hz, and 37 °C.

The overall synthetic strategy and hydrogel design concept are illustrated in Figure 1. Two polymer components were independently prepared and subsequently combined to form the photocleavable hydrogel network. In the first track, gelatin type A was sequentially modified to introduce azide functional groups via NHS ester coupling with NHS-PEG4-azide, yielding gelatin–azide (GA), followed by acetylation of residual primary amines with acetic anhydride to produce acetylated gelatin–azide (AGA). In the second track, 4armPEG-NH_2_ was functionalized with either DBCO-PEG4-NHS ester or PC DBCO-PEG4-NHS ester through NHS ester coupling reactions, yielding 4armPEG-DBCO and 4armPEG-PC-DBCO, respectively. Upon mixing, the azide groups of AGA and the DBCO groups of 4armPEG-PC-DBCO undergo strain-promoted azide–alkyne cycloaddition (SPAAC) spontaneously under physiological conditions without any photoinitiator or light source, forming a stable crosslinked hydrogel network. Exposure of the resulting PC hydrogel to 365 nm UV light cleaves the o-nitrobenzyl linker embedded within the crosslinking junction, leading to network degradation and enabling the release of encapsulated substances. The chemical modifications at each step were verified by FT-IR, ^1^H NMR, and UV-Vis spectroscopy (Figure 2 and Figure 3), the optimal hydrogel formulation was determined by rheological analysis (Figure 4), and the photocleavage behavior was evaluated by visual observation and time-sweep rheology (Figure 5).

### 3.1. Synthesis and Chemical Characterization of AGA

The chemical modifications of gelatin type A were verified by FT-IR and ^1^H NMR spectroscopy at each step (Figure 2).

In the FT-IR spectra, unmodified gelatin exhibited no absorption in the 2050–2150 cm^−1^ region. Following NHS-PEG4-azide coupling, a characteristic azide stretching band appeared at approximately 2100 cm^−1^ in GA, confirming the introduction of azide groups onto the gelatin backbone. This band was retained in AGA, indicating that the subsequent acetylation step did not disrupt the incorporated azide functionality. Additionally, the amide II band at approximately 1550 cm^−1^, present in all three spectra, reflects the amide bond character of the gelatin backbone and the additional amide bonds formed through the NHS ester coupling reaction.

In the ^1^H NMR spectra, the signal at approximately 3 ppm (box i), attributed to the methylene protons of lysine residues adjacent to the primary amine group, showed a progressive reduction from gelatin to GA and further to AGA, reflecting the stepwise consumption of free amine groups through azide conjugation and acetylation. The peak at approximately 1.9 ppm (box ii), which is present in unmodified gelatin owing to amino acid residue signals, became significantly more prominent in AGA, consistent with the contribution of methyl protons from the acetyl groups introduced by acetic anhydride treatment. Collectively, the FT-IR and ^1^H NMR results qualitatively confirm successful azide functionalization and subsequent acetylation of residual amine groups. Quantitative determination of the degrees of azide substitution and acetylation was not performed, as the heterogeneous nature of gelatin results in broad and overlapping spectroscopic signals that limit reliable quantification by conventional FT-IR and ^1^H NMR analyses [11].

### 3.2. Synthesis and Chemical Characterization of 4armPEG-DBCO and 4armPEG-PC-DBCO

The chemical modifications of 4armPEG-NH_2_ were verified by FT-IR, ^1^H NMR, and UV-Vis spectroscopy (Figure 3).

In the FT-IR spectra, unmodified 4armPEG-NH_2_ showed no amide-related absorptions, as expected for a PEG backbone bearing only terminal primary amine groups. Following NHS ester coupling, both 4armPEG-DBCO and 4armPEG-PC-DBCO exhibited a secondary amide N–H stretching band at 3340 cm^−1^, an amide I C=O stretching band at 1650 cm^−1^, and an amide II N–H bending band at 1550 cm^−1^, collectively confirming successful amide bond formation at the PEG amine termini. 4armPEG-PC-DBCO additionally displayed two bands absent in 4armPEG-DBCO: a C=O stretching band at 1760 cm^−1^ attributable to the carbonate linkage within the PC linker structure, and an N=O asymmetric stretching band at 1350 cm^−1^ corresponding to the nitro group of the o-nitrobenzyl moiety. These two bands together confirm the successful incorporation of the PC linker in 4armPEG-PC-DBCO and clearly distinguish it from 4armPEG-DBCO.

In the ^1^H NMR spectra, 4armPEG-NH_2_ showed no signals in the aromatic region (Box i, ~7–8.5 ppm) or at approximately 5 ppm (Box ii). Both 4armPEG-DBCO and 4armPEG-PC-DBCO exhibited multiple aromatic proton signals in Box i, confirming conjugation of the DBCO moiety to the PEG backbone. 4armPEG-PC-DBCO displayed a more complex signal pattern in this region compared to 4armPEG-DBCO, consistent with the additional aromatic protons contributed by the o-nitrobenzyl group of the PC linker. In Box ii (~4.5–5.5 ppm), both modified PEGs showed a signal corresponding to the aliphatic CH proton within the DBCO ring structure, with 4armPEG-PC-DBCO displaying additional signal intensity attributable to the benzylic CH proton of the o-nitrobenzyl group.

UV-Vis spectroscopy confirmed the presence of the DBCO chromophore in both modified PEGs through characteristic absorption peaks at approximately 290 nm and 310 nm, which were absent in 4armPEG-NH_2_. Both 4armPEG-DBCO and 4armPEG-PC-DBCO exhibited these characteristic peaks, confirming DBCO incorporation in both derivatives. The DBCO content of 4armPEG-DBCO was quantified using the molar extinction coefficient of DBCO at 309 nm (ε_309_ = 12,000 M^−1^cm^−1^), yielding a baseline-corrected absorbance of 1.24 and a calculated DBCO concentration of 1.03 × 10^−4^ mol/L. Given the 4armPEG-NH_2_ concentration of 5.0 × 10^−5^ mol/L (0.5 mg/mL, M_n = 10 kDa), this corresponds to approximately 2.1 DBCO groups per PEG molecule, indicating a conjugation efficiency of ~52% relative to the four available amine termini. Although the UV-Vis spectrum of 4armPEG-PC-DBCO exhibits a comparable absorbance profile in the DBCO-associated region, direct quantification of DBCO substitution was not possible because the absorption bands of the o-nitrobenzyl photocleavable group (typically ~300–350 nm) substantially overlap with the characteristic DBCO absorption bands (~290 and 310 nm), precluding reliable spectral deconvolution and accurate determination of DBCO content [12,13,14]. Nevertheless, the similar spectral features, together with FT-IR and ^1^H NMR characterization, indicate successful incorporation of DBCO into the photocleavable derivative.

### 3.3. Hydrogel Formation and Rheological Optimization

The viscoelastic properties of hydrogels formed by combining 4% (*w*/*v*) AGA with varying concentrations of 4armPEG-PC-DBCO were evaluated by oscillatory frequency sweep rheology at 37 °C (Figure 4). Gel formation was defined as conditions under which G′ consistently exceeded G″ across the measured frequency range of 0.1–10 Hz.

At 4armPEG-PC-DBCO concentrations of 0.25% and 0.5% (*w*/*v*), G″ exceeded G′ across essentially the entire frequency range, with G′ values at approximately 1 Hz of 0.00023 Pa and 0.36 Pa, respectively (Figure 4a,b). The highly erratic G′ values observed in these formulations are characteristic of viscous liquid behavior, indicating that crosslinker concentrations below 1% were insufficient to form a percolating network under these conditions.

In contrast, hydrogels formed at 1%, 2%, and 4% (*w*/*v*) 4armPEG-PC-DBCO exhibited G′ values consistently and substantially greater than G″ across the full frequency range, with G′ displaying a plateau-like response characteristic of an elastically crosslinked network (Figure 4c–e). At approximately 1 Hz, G′ values were 20.0 Pa, 44.6 Pa, and 14.4 Pa for the 1%, 2%, and 4% formulations, respectively. The 2% formulation yielded the highest G′, while the 4% formulation showed a marked decrease in G′ relative to 2%, indicating a non-monotonic dependence of network stiffness on crosslinker concentration. This behavior may reflect less efficient network formation at elevated 4armPEG-PC-DBCO concentrations, potentially arising from a deviation from the optimal balance of reactive azide and DBCO groups and from increased network heterogeneity. Based on these findings, 4% (*w*/*v*) AGA and 2% (*w*/*v*) 4armPEG-PC-DBCO were selected as the optimal formulation for subsequent photocleavage characterization.

### 3.4. UV-Triggered Photocleavage Behavior

The photocleavage behavior of the PC hydrogel (4% AGA/2% 4armPEG-PC-DBCO) under 365 nm UV irradiation was evaluated by visual observation and time-sweep rheology (Figure 5).

Macroscopic changes in hydrogel morphology were recorded at 30 s intervals during UV irradiation (Figure 5a). Prior to irradiation, the hydrogel maintained a well-defined dome shape on the rheometer plate. The gel structure remained largely intact through 60 s of irradiation, after which progressive structural deformation became evident at 90 s. By 120 s, substantial loss of structural integrity was observed, and the sample after complete UV exposure showed near-complete transition to a liquid state. These observations confirm that 365 nm UV irradiation induces macroscopic degradation of the PC hydrogel network through photocleavage of the o-nitrobenzyl crosslinker.

The mechanical response to UV irradiation was further characterized by in situ time-sweep rheology (Figure 5b,c). In the absence of UV irradiation, G′ remained stable at approximately 20–25 Pa throughout the 200 s measurement period, consistently exceeding G″ by more than one order of magnitude, confirming the structural integrity of the PC hydrogel under non-irradiated conditions (Figure 5b). Upon initiation of UV irradiation at 60 s, G′ declined rapidly from approximately 12 Pa to values approaching the instrument detection limit within approximately 20 s of UV onset, reaching crossover with G″ at around 80 s (Figure 5c). Following this transition, both G′ and G″ remained at low, erratic values throughout the remainder of the measurement, with no recovery of the G′ > G″ relationship, indicating irreversible disruption of the crosslinked network. Taken together, the visual and rheological data demonstrate that UV-induced photocleavage of the o-nitrobenzyl linker at the crosslinking junctions leads to rapid and irreversible gel-to-sol transition of the PC hydrogel.

## 4. Discussion

In this study, we developed a photo-cleavable gelatin–PEG hybrid hydrogel in which the o-nitrobenzyl (o-NB) photocleavable linker was integrated into the PEG crosslinker arm rather than directly conjugated to a payload molecule. Systematic characterization of both precursor components—acetylated gelatin–azide (AGA) and 4armPEG-PC-DBCO—confirmed successful incorporation of the intended functional groups, and the resulting hydrogel demonstrated UV-triggered dissolution upon 365 nm irradiation. Together, these results validate the core design strategy of incorporating the photocleavable moiety within the hydrogel crosslinker to enable UV-triggered network degradation.

The gelatin–PEG hybrid backbone was selected to leverage complementary properties of each component. Gelatin retains the integrin-binding RGD sequences and matrix metalloproteinase (MMP)-cleavage sites of its collagen precursor, providing intrinsic bioactivity that supports cell adhesion and remodeling [15,16]. PEG, by contrast, contributes mechanical robustness and resistance to non-specific protein adsorption, and its multi-arm architecture enables precise control over crosslink density and network topology [17,18]. Crosslinking was accomplished via strain-promoted azide–alkyne cycloaddition (SPAAC), a bioorthogonal reaction that proceeds efficiently under physiological conditions (aqueous buffer, 37 °C) without catalysts, photoinitiators, or exogenous reagents [19,20]. Frequency sweep measurements confirmed elastic-dominant behavior (G′ > G″) across the tested range, consistent with a covalently crosslinked network rather than a physical gel. The measured storage modulus (~44 Pa) indicates the formation of a relatively soft hydrogel network. While this formulation was not designed to replicate the mechanical properties of any specific tissue, the photocleavable nature of the system may be advantageous for applications requiring temporary structural support followed by user-controlled degradation. Such dynamic degradation could facilitate cell migration, tissue remodeling, or controlled scaffold removal, depending on the intended application. Further optimization of polymer composition, crosslinking density, and network architecture will be required to tailor the mechanical properties for specific tissue engineering applications.

A key prerequisite for selective SPAAC crosslinking was the capping of residual primary amines on gelatin–azide. In the GA intermediate, NHS-PEG4-azide reacts preferentially with primary amines; however, unconjugated lysine residues remain and could interfere with NHS ester–amine chemistry during subsequent fabrication steps or in biological environments. Acetylation with acetic anhydride at mildly alkaline pH (8.0–8.5) was employed to cap residual primary amines by converting them to neutral acetamides, thereby minimizing competing reactive amine functionalities and making the azide group the predominant reactive handle available for SPAAC crosslinking. The importance of this step is evidenced by the FT-IR and ^1^H NMR spectra, which show the emergence of acetamide-associated signals in AGA relative to GA, confirming successful capping. This design choice minimizes competing side reactions and provides a chemically well-defined precursor for reproducible hydrogel formation.

The central innovation of this system is the placement of the o-NB photocleavable moiety within the crosslinker arm of 4armPEG-PC-DBCO, rather than covalently linking it to a drug or bioactive molecule. Prevailing photo-triggered release strategies typically require direct conjugation of the photocleavable linker to the payload, which necessitates chemical modification of each molecule of interest and risks altering its biological activity [5,7]. In the present approach, photocleavage-driven network dissolution is expected to permit the release of physically entrapped molecules without requiring their chemical modification. This feature may be particularly advantageous for biologically sensitive payloads such as proteins, growth factors, peptides, or MSC-derived secretome, whose activity may be affected by direct chemical conjugation strategies. Moreover, the rapid loss of network integrity observed following UV irradiation suggests that release may potentially be triggered using relatively short irradiation periods, which could help minimize light exposure of encapsulated bioactive molecules. However, direct loading and release studies will be required to experimentally validate these potential advantages.

Incorporation of the o-NB moiety into 4armPEG-PC-DBCO was confirmed primarily by FT-IR and ^1^H NMR analyses, which showed the carbonate (1760 cm^−1^) and nitro (1350 cm^−1^) bands and the additional aromatic and benzylic proton signals of the o-nitrobenzyl group that were absent in 4armPEG-DBCO. In the UV-Vis spectra, the o-nitrobenzyl chromophore absorbs in the same region as the DBCO bands (~290 and 310 nm) [21], so the two contributions could not be spectrally resolved; nevertheless, the comparable absorption profile of 4armPEG-PC-DBCO relative to 4armPEG-DBCO in this region is consistent with successful incorporation of both moieties. This approach is conceptually aligned with photodegradable hydrogel platforms in which o-NB-functionalized PEG crosslinkers enable spatially patterned network erosion [22], but differs in that it employs SPAAC bioorthogonal chemistry and a gelatin-based biopolymer backbone, expanding its potential compatibility with cell-laden systems.

UV irradiation at 365 nm triggered rapid degradation of the hydrogel, as confirmed by both macroscopic dissolution and the time-dependent decrease in G′ observed during in situ rheological monitoring. The 365 nm wavelength was deliberately chosen for its favorable cytocompatibility profile; at this wavelength, DNA damage in mammalian cells is substantially lower than at shorter UV wavelengths (e.g., 254 or 302 nm), and prior studies have demonstrated high cell viability following 365 nm exposure within the doses required for o-NB cleavage [23,24]. The photocleavage of o-NB linkers is reported to generate o-nitrosobenzaldehyde-derived byproducts, which have been noted as potential sources of cytotoxicity in certain contexts [5]; Although preliminary Live/Dead analysis did not reveal detectable cytotoxic effects under the conditions investigated (Figure S1), careful optimization of irradiation dose, hydrogel formulation, and photocleavage conditions will be important in future studies to balance degradation efficiency with biological compatibility. Nevertheless, the prompt G′ collapse observed upon UV initiation at 60 s in the time sweep experiment demonstrates that network degradation is rapid and well-controlled, supporting the utility of this system for on-demand, temporally controlled release applications. In future studies, localized or patterned light exposure strategies may further extend the platform toward spatially controlled degradation and release applications.

This study focused on material-level characterization of the hydrogel platform, and several aspects remain to be addressed. Direct experimental validation of payload release has not yet been performed, and the loading efficiency and release kinetics of model drugs or bioactive molecules remain to be quantified. Although preliminary cytocompatibility was demonstrated, more comprehensive biological evaluation and in vivo studies will be required before biomedical application. In addition, application-specific characterization of swelling behavior, long-term stability under physiological conditions, degradation in PBS, and injectability will be important for future translation of the platform. The UV irradiation parameters (intensity, exposure duration, penetration depth) will need systematic optimization, particularly for three-dimensional or tissue-embedded constructs where light attenuation is significant. Furthermore, the potential of near-infrared (NIR)-to-UV up conversion strategies or two-photon excitation to extend the addressable tissue depth warrants future investigation [5,6]. Collectively, the results presented here establish a chemically well-defined, modular platform in which UV-selective hydrogel degradation is achieved independently of payload identity, providing a foundation for the development of versatile on-demand release systems and future degradable hydrogel platforms with application-specific mechanical properties.

## 5. Conclusions

In summary, we successfully designed and characterized a photo-cleavable gelatin–PEG hybrid hydrogel in which bioorthogonal SPAAC crosslinking and UV-responsive degradation are unified in a single system by integrating an o-nitrobenzyl photocleavable linker into the PEG crosslinker arm. The stepwise synthesis of AGA and 4armPEG-PC-DBCO was confirmed by spectroscopic characterization, and the resulting hydrogel exhibited well-defined elastic network behavior under physiological conditions and underwent rapid dissolution upon 365 nm UV irradiation. By decoupling the photo-trigger from the payload, this design eliminates the need to directly conjugate a photocleavable linker to the encapsulated molecule and is therefore potentially compatible with a wide range of drugs, proteins, and bioactive factors. Although further studies on payload loading and release, comprehensive biocompatibility assessment, and application-specific hydrogel characterization remain necessary, the material-level results presented here establish this system as a promising and modular platform for on-demand release of bioactive molecules. Future optimization of the mechanical properties may further enable applications that require temporary structural support followed by controlled degradation, which we aim to explore in subsequent studies.

## Data Availability

The original contributions presented in this study are included in the article/Supplementary Materials. Further inquiries can be directed to the corresponding authors.

## Supplementary Materials

The following supporting information can be downloaded at: https://www.mdpi.com/article/10.3390/ma19122625/s1.

## Abbreviations

The following abbreviations are used in this manuscript:

AGA	Acetylated gelatin–azide
ATR	Attenuated total reflectance
DBCO	Dibenzocyclooctyne
DMSO	Dimethyl sulfoxide
FT-IR	Fourier-transform infrared spectroscopy
GA	Gelatin–azide
MMP	Matrix metalloproteinase
MWCO	Molecular weight cut-off
NHS	N-hydroxysuccinimide
NIR	Near-infrared
NMR	Nuclear magnetic resonance
o-NB	o-Nitrobenzyl
PBS	Phosphate-buffered saline
PC	Photocleavable
PEG	Poly(ethylene glycol)
RGD	Arg-Gly-Asp
SPAAC	Strain-promoted azide–alkyne cycloaddition
UV	Ultraviolet
UV-Vis	Ultraviolet-visible spectroscopy
4armPEG-DBCO	Four-arm poly(ethylene glycol)-dibenzocyclooctyne
4armPEG-NH_2_	Four-arm poly(ethylene glycol) amine
4armPEG-PC-DBCO	Four-arm poly(ethylene glycol)-photocleavable-dibenzocyclooctyne
