# Supplementary material for: Crosslinker-Integrated Photocleavable Gelatin–PEG Hydrogel via Bioorthogonal SPAAC Chemistry for UV-Triggered On-Demand Degradation
Source: Materials (Basel). 2026 Jun 18;19(12):2625. doi: 10.3390/ma19122625 (PMC13304095; doi:10.3390/ma19122625)
Supplement: Supplementary file 1 [file materials-19-02625-s001.zip › materials-4347896-supplementary.pdf]

Supplementary Information

Crosslinker-Integrated Photocleavable Gelatin–PEG Hydrogel via Bioorthogonal SPAAC Chemistry for UV-Triggered On-Demand Degradation

Yeon Tae Kang, Gayeon Pyo, Karthika Muthuramalingam * and Hyun Jong Lee *

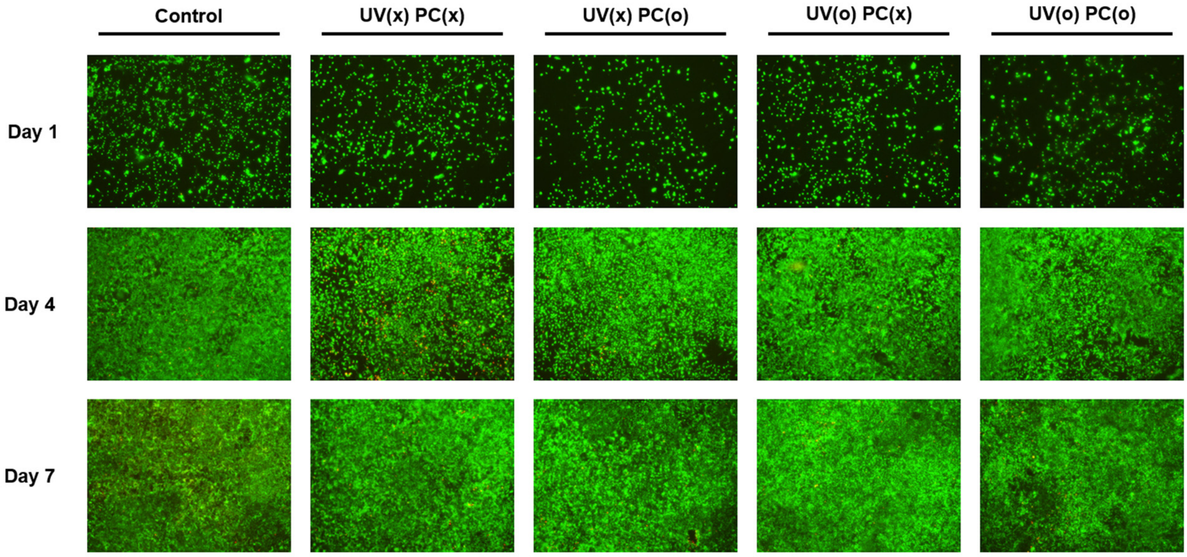

Figure S1. Live/Dead analysis of NIH/3T3 cells exposed to hydrogels with or without a photocleavable linker under UV irradiation conditions. Representative Live/Dead fluorescence images obtained on Days 1, 4, and 7 following treatment. NIH/3T3 cells were pre-seeded in tissue culture plates, and hydrogel samples were placed in cell culture inserts positioned above the cells. UV irradiation was applied from the top under the same conditions used for photocleavage experiments. Experimental groups included Control (no hydrogel), UV(x) PC(x), UV(x) PC(o), UV(o) PC(x), and UV(o) PC(o), where UV(o) and UV(x) indicate the presence and absence of UV irradiation, respectively, and PC(o) and PC(x) indicate the presence and absence of the photocleavable (PC) linker. Live cells are stained green and dead cells are stained red. Predominantly green fluorescence with minimal red staining was observed in all groups throughout the 7-day culture period. Comparable cell viability was maintained regardless of UV exposure or incorporation of the photocleavable linker, indicating that neither the applied UV irradiation nor products generated following photocleavage induced detectable cytotoxicity under the experimental conditions.

Materials and Methods

Cell culture and Live/Dead assay

NIH/3T3 fibroblasts (KCLB No. 21658) were obtained from the Korean Cell Line Bank (Seoul, Republic of Korea) and cultured in DMEM supplemented with 10% FBS and 1% penicillin–streptomycin at 37°C under 5% CO_2. Cells were seeded in 24-well plates and allowed to attach for 24 h. Hydrogel samples (with or without the PC linker) were prepared in cell culture inserts positioned above the cells. For UV groups, 365 nm irradiation was applied from the top under the same conditions used for the photocleavage experiments. On Days 1, 4, and 7, cells were stained using a Live/Dead viability kit according to the manufacturer's instructions (LIVE/DEAD™ Viability/Cytotoxicity Kit (Invitrogen, USA)). Live (green) and dead (red) cells were visualized for each group.
